# Under Ideal Conditions

**Published:** 1921-08-20

**Authors:** 


### Under Ideal Conditions.

BY A SOCIAL WORKER.

The Hospital issue of July 30 bore on its cover the familiar statement that Bournville Cocoa is made "under ideal conditions." It is certainly a challenging statement. "Whose ideal?" one is tempted to ask as a first of many questions. Go there and the beauty of the place grips your heart at once. The lie of the ground, sloping both ways to a lovely brook, fully utilised for fishing and landscape-making, the fine trees and fresh air, are Nature's contribution. The handsome school, with a certain home-likeness in its architecture, its frescoed hall and cheerful classrooms, which refuse unwieldy numbers, and its carillon copied from Bruges, may strike you first. Then the houses of all sizes; no ugly one, and none without garden; the rest-house presented by employees as Mr. and Mrs. Cadbury's silver-wedding gift; yet before you reach the beautiful buildings and grounds of the "factory in a garden," you have learnt that large numbers of the workpeople prefer to make their homes in dingy Birmingham, coming out and returning there daily.

Some forty years of open-minded experiment have gone to the making of conditions by people who have had an ever-rising ideal in view for the lives of workers, who now number 9,000, and whose occupation is by no means merely the making of cocoa and chocolates. Twenty trades unions are in touch with the works council; 400 girls make cardboard boxes; the milk of 3,000 cows has every day to be brought and dealt with; about 1,000,000 meals are prepared and served in a year, apart from any functions—the annual summer party, for instance, may number over 8,000.

A few details of the healthful organisation of life will most interest readers of The Hospital. Perhaps the smaller details, indeed, have most interest. It is, for instance, a common thing to find butchers' men suffering from rupture and other troubles as the result of lifting heavy carcases. At Bournville certain employees whose work makes the lifting of weight necessary are given special drill and training in the right use of muscles. So also with regard to accidents. A whole-time expert is in charge of all machinery, and fresh devices are added when needed, but many of these have been thought out by the workers themselves, and prizes have been awarded. The constantly recurring difficulty that workers will not use safety devices is thus combated. Physical training is compulsory for the young people, and an extremely wise regulation provides that for a lesson lasting half an hour a whole hour's time off is given, so as to allow of unhurried going and returning.

Young people entering at the age of fourteen are examined by doctor and dentist. They are going not merely to learn a good trade, but also they will have four years of continued education; they should, therefore, be reasonably good raw material. The present official scheme for fourteen- to sixteen-year-olds is no novelty at Bournville; up to eighteen the opportunities continue, with many encouraging devices, such as repayment by the firm of fees to outside classes on proof of attendance and progress. Domestic training is emphasised for the girls, who take their fourth year's course at an ordinary cottage in the village. As 75 per cent. marry in their twenties the gain is certainly not primarily to the firm. Gardening and ambulance classes are good aids to health; still more so is the swimming, which is compulsory for both girls and boys. For games the firm employ five professional teachers. There are rest-rooms for temporarily ailing girls; there is a convalescent home, with twenty-one beds, at Bromyard; every year there are outdoor summer camps for both girls and boys. Hot baths are available at any time, and for girls there are welcome hair-drying appliances. For dinner girls have a dining-room holding 2,000, and those who bring their own dinners can heat them in a closed cupboard. A perennial fountain of hot water is available for a cup of tea. Here the tables collapse and make backs to seats, so that the organ and other music may be comfortably listened to. There is a lending library; there is a works magazine; there are recreation grounds, with courts for all games both for girls and men. In the midst of these, near a lovely little Italian garden, stands what looks like a Flemish Béguinage, a double row of almshouses, with one in the centre for a matron, who visits each inmate daily. At present few of the dwellers are employees, for the excellent reason that the wisely contributory pension earned after full service is beyond the income maximum permitted. Now with all these costly advantages to be provided, working hours per week are forty-eight for men, forty-two for women.

Visitors from all countries come to Bournville, and cannot fail to carry away a sense of admiration and hope. Its death-rate is 3 per 1,000; its life—well, is *life*, and not mere existence. Are not these something like "ideal conditions"?

